# Exploring Non-antibiotic Interventions for Preventing Urinary Tract Infections During Pregnancy: A Systematic Review

**DOI:** 10.7759/cureus.93874

**Published:** 2025-10-05

**Authors:** Shivani Srivastava, Shreekar Roddam, Deepshikha Srivastava, Abdulmumini Shehu, Khyathi krishna Gogineni, Ruchit Singla, Ahmed Owolabi, Basma Alleelwa, Aliyu O Olaniyi, Olabisi P Lawal

**Affiliations:** 1 General Medicine, Stepping Hill Hospital, Stockport, GBR; 2 General Practice, Swansea Bay University Health Board, Swansea, GBR; 3 General Surgery, Barnsley Hospital, Rotherham, GBR; 4 Stroke Medicine, Stepping Hill Hospital, Stockport, GBR; 5 Medicine, Stepping Hill Hospital, Stockport, GBR; 6 Acute Medicine, Stockport NHS Foundation Trust, Stockport, GBR; 7 Acute Medical Unit, Stepping Hill Hospital, Stockport, GBR; 8 Geriatrics, Stepping Hill Hospital, Stockport, GBR; 9 Medical Laboratory Science, University of Benin, Benin City, NGA

**Keywords:** antimicrobial resistance, ascorbic acid, cranberry, non-antibiotic interventions, pregnancy, urinary tract infections

## Abstract

Urinary tract infections (UTIs) are a common health concern in pregnancy and often require antibiotic therapy. Antimicrobial resistance (AMR) resulting from antibiotic misuse is a major public health challenge, and non-antibiotic interventions (NAIs) may provide a safer means of prevention. This systematic review evaluated non-antibiotic strategies for preventing UTIs during pregnancy as a way to reduce antibiotic prescribing and combat AMR. The review aims to evaluate the role of NAIs in preventing UTIs during pregnancy, with a focus on their potential to reduce antibiotic prescribing and AMR within the NHS care system. A systematic search of Ovid Medline, Web of Science, and Scopus identified studies that compared NAIs to a placebo, usual treatment, or no intervention. Eligible studies included randomised controlled trials, cohort studies, and other relevant designs. Study quality was assessed using established appraisal tools, and a narrative synthesis was conducted due to intervention heterogeneity. Eight studies met the inclusion criteria, examining cranberry products, Mediterranean diet, OM-89 (an oral immunostimulant also known as Uro-Vaxom), ascorbic acid, and knowledge-based interventions. Evidence from seven studies suggests that NAIs may reduce UTI incidence, but the findings are limited, and further research is needed to confirm their effectiveness during pregnancy. The review concludes that NAIs, particularly cranberry products and the Mediterranean diet, may reduce UTI incidence during pregnancy and offer a promising strategy to limit antibiotic use and AMR. However, given the methodological limitations and low quality of available evidence, further high-quality research is required to establish the safety, efficacy, and cost-effectiveness of these therapies in pregnant women.

## Introduction and background

One of the most common infections in pregnancy is urinary tract infections (UTIs), which affects a large percentage of women [1]. Some of the complications of UTIs during pregnancy are preterm birth, low birth weight, and maternal morbidity [2,3]. Antibiotics are conventionally used to treat most UTIs in pregnancy, but their overuse and misuse have resulted in antimicrobial resistance (AMR), which is a major public health concern [4]. AMR is essentially caused by pathogens such as *Escherichia coli *becoming resistant to common medications, which makes treating infections hard [5]. Because only a few antibiotics are safe to use in pregnancy, as some antibiotics can harm the foetus, AMR to these antibiotics can be really dangerous for pregnant women [1]. As per the National Institute for Health and Care Excellence (NICE) 2018 clinical guidelines (NG109), UTI treatment in pregnancy is limited to nitrofurantoin and cefalexin [6]. With the recent rise in AMR to these antibiotics, future treatment options are a concerning issue. The overconsumption and overprescription of antibiotics, particularly broad-spectrum antibiotics, have increased resistance [7]. It is hence important to explore approaches such as non-antibiotic interventions (NAIs) to prevent UTIs in pregnancy. NAIs aim to prevent/minimise UTIs, hence reducing AMR. Examples of NAIs include cranberry products, Mediterranean diet, OM-89, probiotics, ascorbic acid, lifestyle adjustments, and knowledge-based intervention [8,9]. Cranberry contains proanthocyanidin, which inhibits the adhesion of bacteria to the urinary tract [10]. The Mediterranean diet includes olive oil and pistachios, which have anti-inflammatory and antibacterial properties [11]. OM-89 is an oral immunostimulant derived from bacterial lysates that has shown potential in reducing recurrent UTIs during pregnancy, though current evidence is limited and requires confirmation through larger, well-designed trials [8]. Knowledge-based interventions for UTI prevention involve educating pregnant women on proper hygiene and behaviours that reduce infection risk, such as genital hygiene, regular urination, adequate hydration, and avoiding irritants. These strategies empower women to actively prevent UTIs, especially in settings with limited access to medical prophylaxis [8]. The evidence around the efficacy of NAIs is limited and mostly from low-quality studies [12]. There are several studies on NAIs, such as cranberry products and probiotics, but they are on non-pregnant women [13,14]. Hence, there is a gap in the literature surrounding NAIs in pregnancy to prevent UTIs as a means to reduce AMR.

Most studies on NAIs for UTI prevention have been conducted on the general population and do not focus on pregnant women. There are very few trials that have been conducted to compare the efficacy of NAIs vs antibiotics in pregnant women to prevent UTIs. The study design, type of intervention, or sample sizes of these trials are inappropriate to draw appropriate evidence about the efficacy of NAIs for pregnant women. There is evidence surrounding the Mediterranean diet lowering UTIs, but the data are variable and equivocal among the population [11,15]. There is insufficient data on how NAI affects the prescribing of antibiotics. Moreover, studies on NAIs focus on prevention/ reduction of UTIs, but do not assess their use in reducing the use of antibiotics for treatment as a means of tackling AMR [16]. There are very few studies that have assessed the cost-effectiveness of NAIs in pregnant women, hence making it difficult for policy makers to incorporate this into guidelines or routine practice. This systematic review amalgamates evidence from various studies done on NAIs to prevent UTIs in pregnant women to fill these gaps. This review highlights how NAIs can be used in clinical practice for pregnant women by assessing their safety, efficacy, and antibiotic reduction potential as a means to tackle AMR. Further, this review identifies areas that require further research to bridge the gap in knowledge and strengthen evidence surrounding NAIs in pregnancy.

The aim of this systematic review is to bridge this gap in research by inspecting the role of NAIs in clinical practice in preventing UTIs during pregnancy as a potential strategy to limit antibiotic resistance by reducing prescribing. The objective of this literature review in a systematic manner is to identify NAIs that can be used to prevent the occurrence of UTIs during pregnancy, critically appraise the evidence for the use of these interventions in pregnancy, perform a rapid review of the included studies to provide an overview of the potential of non-antibiotic prophylaxis to reduce incidence of UTIs, and summarise the results in the context of National Health Service (NHS) care delivery, including impact on AMR.

## Review

Methodology

Study Design

A systematic review was conducted to assess the effectiveness of various non-antibiotic interventions in preventing UTIs during pregnancy. The review adhered to the Preferred Reporting Items for Systematic Reviews and Meta-Analyses (PRISMA) guidelines to ensure a transparent, rigorous, and reproducible process. All stages of the review, including study identification, selection, data extraction, and quality assessment, were carried out systematically to provide a comprehensive synthesis of the available evidence.

Search Strategy

A comprehensive search strategy was developed to identify studies addressing the use of NAIs in preventing UTIs during pregnancy. The strategy included the use of key terms and synonyms, MeSH (Medical Subject Headings) terms, and filters. The search strategy was adapted for each database to optimize results.

Key terms: The search strategy incorporated key terms across four main concepts. For pregnancy, terms used included “pregnancy”, “pregnant women”, and “gestation”. Prevention-related terms comprised the following: “prevention”, “prophylaxis”, “reduce risk”, and “non-antibiotic prevention”. UTIs were searched using these terms: “urinary tract infection”, “UTI”, “cystitis”, “pyelonephritis”, “asymptomatic”, and “bacteriuria”. Non-antibiotic interventions were identified through terms such as “cranberry”, “Mediterranean diet”, “OM-89”, “ascorbic acid”, “probiotics”, “dietary interventions”, and “education”. These terms were combined to ensure comprehensive retrieval of relevant studies evaluating non-antibiotic strategies for UTI prevention in pregnancy.

Example of search query (Ovid Medline): The search strategy combined key concepts related to pregnancy, prevention, UTIs, and non-antibiotic interventions. Pregnancy was captured using the terms "exp Pregnancy/" or "pregnant.mp.", while prevention was identified through "prevention.mp." or "exp Primary Prevention/". Urinary tract infections were searched using "urinary tract infections.mp." or "exp Urinary Tract Infections/". To capture the interventions of interest, terms such as "cranberry.mp.", "Mediterranean diet.mp.", "OM-89.mp.", and "ascorbic acid.mp." were used. Finally, these concepts were combined with the Boolean operator #1 AND #2 AND #3 AND #4 to ensure the retrieval of studies addressing all components of the review question. The strategy was tailored for each database, and results were restricted to studies published in English. Citation chaining and manual searches were also performed to identify additional relevant studies.

Inclusion Criteria

This systematic review was done on studies involving pregnant women at any stage of gestation, whether nulliparous, primiparous, or multiparous. The interventions considered for the study were NAIs aimed at preventing UTIs during pregnancy, including cranberry products, the Mediterranean diet, OM-89 (a bacterial extract), ascorbic acid, and educational strategies regarding UTI prevention. These interventions were compared against placebo, standard care, pre-intervention data, or varying levels of adherence to the intervention. The primary outcome assessed was the incidence of UTIs during pregnancy, including asymptomatic bacteriuria, symptomatic bacteriuria, cystitis, and pyelonephritis. Eligible study designs included in the study were randomised controlled trials (RCTs), cohort studies, pre-post studies, and quasi-experimental studies that reported on UTI incidence with comparative data between NAI and control groups. Studies conducted in any healthcare setting where pregnant women received antenatal care were considered.

Exclusion Criteria

In this review, some studies were excluded because they did not meet the predefined eligibility criteria. Non-human or in vitro studies, such as those conducted in laboratory settings without human participants, were not included. Additionally, studies published in languages other than English were excluded due to language constraints. Studies involving pregnant women with adverse health conditions, including immunocompromised states or multiple chronic conditions that could confound UTI incidence, were also not considered. Only studies focused on the prevention of UTIs were eligible; therefore, NAIs aimed at treatment rather than prevention were not included. Furthermore, studies that compared NAIs with antibiotic prophylaxis or other antibiotic treatments were excluded. In addition, research that failed to report UTI incidence rates or other relevant outcomes related to UTI prevention was not considered. Finally, editorials, opinion articles, and other non-empirical studies lacking original data were excluded from the review for a uniform analysis.

Information Sources and Study Selection

Relevant studies were identified through a comprehensive search of multiple databases and sources such as Ovid Medline, Web of Science, and Scopus. Grey literature, such as unpublished studies and reports, was not included in this review to focus on peer-reviewed, high-quality evidence and to ensure reproducibility of findings. Study selection was carried out in a two-step process. Firstly, after removing duplicates, the titles and abstracts of all retrieved records were screened to assess their eligibility based on the predefined inclusion and exclusion criteria. Studies that did not meet the criteria were excluded at this stage. Secondly, the full texts of the remaining studies were obtained and evaluated in detail using an eligibility form to ensure consistency in applying the criteria. Studies failing to meet the requirements at this stage were excluded, and the reasons for exclusion were carefully documented. A flowchart of the study selection process, following the PRISMA guidelines, was used to illustrate the screening, inclusion, and exclusion of studies.

Data Extraction

Data extraction from the included studies was performed using a standardized form that captured key information. Extracted data included study characteristics, such as author(s), year of publication, country, study design, setting, and sample size. Participant details, including age, gestational age, and parity, were also recorded. Information on the intervention was collected, specifying the type and dosage of the NAI used, such as cranberry, the Mediterranean diet, or other approaches. Comparator details, including placebo or standard care groups, were noted. Outcomes focused on incidence rates of UTIs, including asymptomatic bacteriuria, cystitis, and pyelonephritis, along with statistical significance and other relevant findings. Results were summarized, and quality assessment data, such as risk of bias ratings and quality scores, were documented. Data were independently extracted by two reviewers, with any discrepancies resolved through discussion with a second reviewer to ensure accuracy and consistency. The data were independently extracted by two reviewers, and discrepancies were resolved through discussion with a third reviewer.

Quality Assessment

The quality and risk of bias of the included studies were assessed using multiple approved tools. The Modified Downs and Black Tool was applied to evaluate overall study quality, focusing on reporting, external and internal validity (bias), and potential confounding factors, with scores categorizing studies as good, fair, or low quality. The Critical Appraisal Skills Programme (CASP) checklists were used to assess the methodological quality of RCTs and cohort studies, examining aspects such as randomisation, blinding, follow-up, and outcome reporting. Furthermore, the Cochrane Risk of Bias (ROB) tools were used, with ROB-2 applied to RCTs and Risk Of Bias In Non-randomised Studies - of Interventions (ROBINS-I) to non-randomized studies, investigating risks of selection, performance, detection, and attrition bias. Together, these evaluations provided an overall assessment of study quality and bias risk, which was used to guide the synthesis of results.

Synthesis of Results and Key Synthesis Steps

Given the heterogeneity of the papers, a narrative synthesis was done instead of a meta-analysis. Studies were categorised by NAI intervention (e.g., cranberry, Mediterranean diet), and their findings were summarised. The results of each included study were summarised, focusing on reported outcomes such as UTI incidence and statistical significance (p-values). Comparisons were made across studies to evaluate the effectiveness of different NAIs, taking into account study quality and risk of bias. The synthesis indicated that NAIs show promise in preventing UTIs during pregnancy, although further research is needed to strengthen the evidence base. These findings were also discussed in the broader context of reducing antibiotic use and combating AMR in pregnant populations. Overall, the synthesis highlighted both the potential of certain interventions for clinical practice and the need for more rigorous research to improve the quality and consistency of evidence in this field.

Results

Search Results

**Figure 1:**
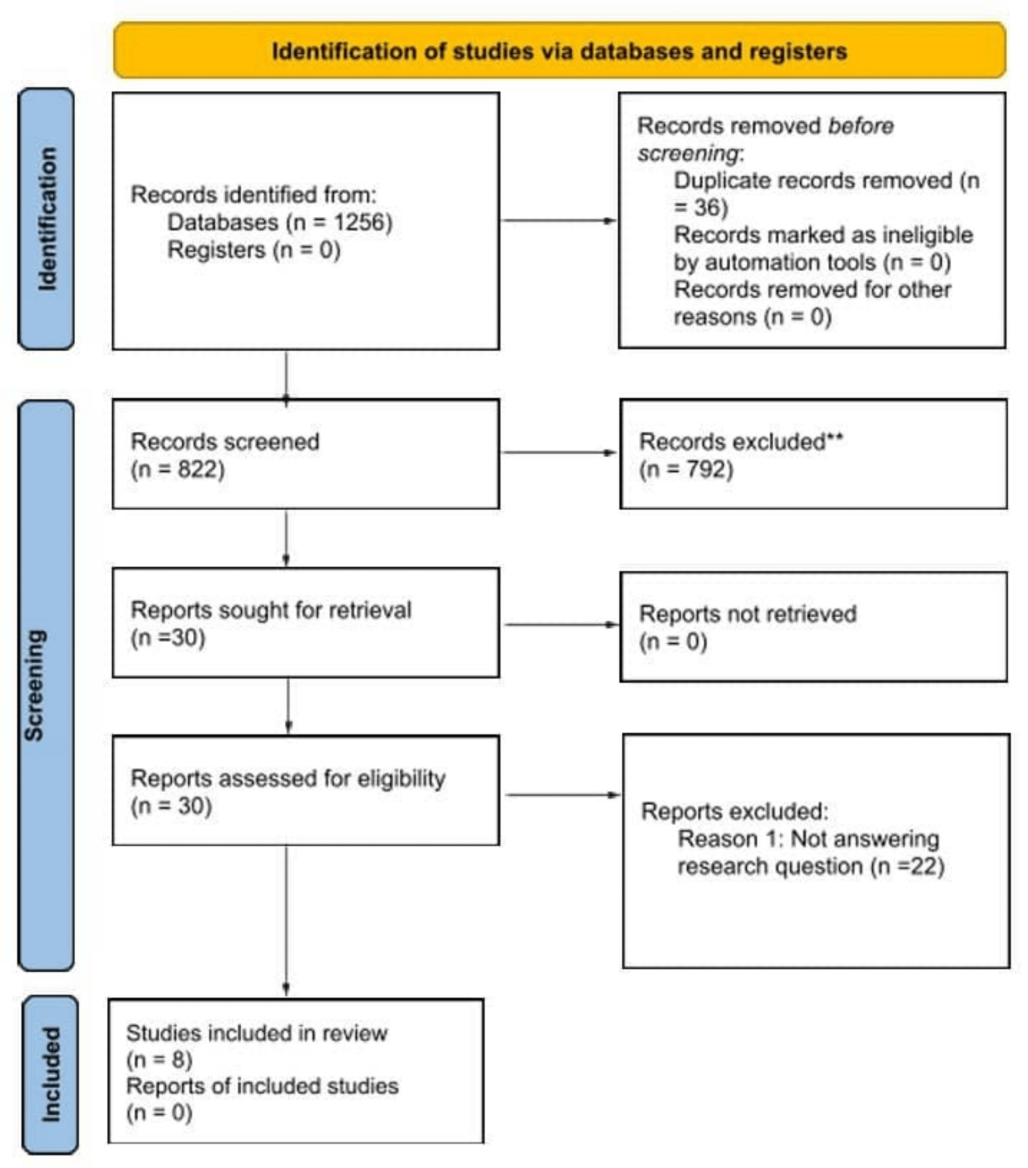
PRISMA 2020 flow diagram for the study selection process PRISMA: Preferred Reporting Items for Systematic Reviews and Meta-Analyses

A comprehensive search across Ovid Medline, Web of Science, and Scopus databases identified a total of 1,256 records. After deduplication, 822 records were screened for eligibility based on titles and abstracts. From these, 792 studies were excluded for failing to meet the inclusion criteria, leaving 30 studies for full-text assessment. Of the 30 full-text articles assessed, eight studies were included in the final review. The selection process is summarized in the PRISMA flow diagram (Figure 1).

Phase Records

A total of 1,256 records were identified through database searching. After the removal of duplicates, 822 records remained and were screened for eligibility. Of these, 30 full-text articles were assessed in detail, and eight studies met the inclusion criteria and were included in the review.

Study Characteristics

The eight studies included in the review varied in terms of study design, sample size, population, and type of NAIs used. A summary of the included studies is provided in Table 1. The review included a total of eight studies with diverse designs: four RCTs, two cohort studies, one pre-post study, and one quasi-experimental study, reflecting the broad methodological approaches used to investigate NAIs for UTI prevention in pregnancy. Sample sizes range from 49 participants in the smallest study to 874 participants in the largest, encompassing both small, targeted trials and larger population-based investigations. The interventions assessed were varied, with three studies evaluating cranberry products in the form of juice or capsules, two studies examining the effects of a Mediterranean diet enriched with extra virgin olive oil and pistachios, one study testing OM-89 (a bacterial extract vaccine), one study assessing ascorbic acid supplementation, and one study exploring knowledge-based interventions through educational strategies for UTI prevention. Across all studies, the primary outcome was the incidence of UTIs, which include asymptomatic bacteriuria, symptomatic bacteriuria, cystitis, and pyelonephritis. These were measured and compared between the intervention and control groups to determine the effectiveness of each intervention.

**Table 1:** Characteristics of the included studies NR: (Not Reported)

Study (Author, Year)	Country	Study Design	Sample Size	Non-antibiotic Intervention	Comparator	Outcome	Study Period
Wing et al. (2008) [17]	USA	RCT	188	Cranberry Juice	Placebo	UTI Incidence	July 2005–2007
Wing et al. (2015) [18]	USA	RCT	49	Cranberry Capsules	Placebo	UTI Incidence	2009–2012
Heitmann et al. (2013) [15]	Norway	Cohort	68,522	Cranberry	No Cranberry	UTI Incidence	1999–2006
Assaf-Balut et al. (2019) [11]	Spain	RCT	697	Mediterranean Diet (Extra Virgin Olive Oil & Pistachios)	Standard Care	UTI Incidence	Jan–Dec 2015
Assaf-Balut et al. (2019) [11]	Spain	Cohort	874	Mediterranean Diet (6 Targets)	Adherence Levels	UTI Incidence	Jan–Dec 2015
Baertschi (2003) [19]	Switzerland	Pre-Post	62	OM-89	Pre-intervention	UTI Recurrence	NR
Navarro et al. (2019) [12]	Philippines	Quasi-Experimental	88	Knowledge Provision (Education & Text Messages)	Pre-intervention/No Education	UTI Incidence	2015
Ochoa-Brust et al. (2007) [20]	Mexico	RCT	110	Ascorbic Acid + Supplements	Supplements Only	UTI Incidence	NR

Risk of Bias Assessment

The quality and risk of bias of the included studies were assessed using the modified Downs and Black tool (Table 2), Critical Appraisal Skills Programme (CASP) checklists, and the Cochrane Risk of Bias tools (ROB-2 and ROBINS-1). Cochrane Risk of Bias Assessment for RCTs is shown in Table 3, and Risk of Bias in Non-RCTs Using ROBINS-1 is shown in Table 4. Two of the four RCTs [17,18] demonstrated low bias due to adequate randomisation, blinding, and participant accountability. Heitmann et al. [15] had a substantial bias risk due to high attrition and poor confounding factor control. Due to unclear blinding and inadequate reporting [11], RCTs experienced moderate bias. Both cohort studies [15,11] had moderate to high bias due to the lack of randomisation, insufficient confounder control, and lack of blinding. Pre-post and quasi-experimental studies [19,12] have substantial bias risk. Unblinded results, absence of randomisation, and uneven follow-up were the issues. Overall, the risk of bias varied across studies, with RCTs generally demonstrating lower risk than non-randomised studies. However, most studies had significant methodological limitations, resulting in a low overall quality of evidence, and caution is warranted when interpreting the conclusions.

**Table 2:** Modified Downs and Black quality assessment scores

Study	Reporting (out of 11)	External Validity (out of 3)	Internal Validity (Bias) (out of 7)	Internal Validity (Confounding) (out of 6)	Power (out of 2)	Total Score (out of 29)	Quality
Wing et al. (2008) [17]	10	3	6	5	1	25	High
Wing et al. (2015) [18]	11	3	6	6	0	26	High
Heitmann et al. (2013) [15]	9	3	2	2	1	17	Fair
Assaf-Balut et al. (2019) [11]	9	3	4	4	1	21	High
Assaf-Balut et al. (2019) [11]	9	3	4	3	0	19	Fair
Navarro et al. (2019) [12]	9	3	0	2	0	14	Low
Baertschi et al. (2003) [19]	7	3	4	1	0	15	Fair
Ochoa-Brust et al. (2007) [20]	8	3	5	3	0	19	Fair

**Table 3:** Cochrane Risk of Bias Assessment for RCTs RCTs: Randomised controlled trials

Study	Selection Bias	Performance Bias	Detection Bias	Attrition Bias	Reporting Bias
Ochoa-Brust et al. (2007) [20]	Low	Low	High	Low	Low
Wing et al. (2008) [17]	Low	Low	Low	Low	Low
Wing et al. (2015) [18]	Low	Low	Low	Low	Low
Assaf-Balut et al. (2019) [11]	Low	Unclear	High	Low	Low

**Table 4:** Risk of Bias in Non-RCTs Using ROBINS-1 ROBINS-1: Risk Of Bias In Non-randomised Studies - of Interventions

Study	Pre-intervention	At Intervention	Post-intervention	Bias due to Confounding	Bias in Outcome Measurement	Risk of Bias
Baertschi et al. (2003) [19]	Low	Low	Moderate	Low	Serious	High
Navarro et al. (2019) [12]	Low	Moderate	Serious	Moderate	Serious	Serious
Assaf-Balut et al. (2019) [11]	Moderate	Moderate	Serious	Low	Serious	Moderate
Heitmann et al. (2013) [15]	Low	Low	Critical	Serious	Critical	Critical

Synthesis of Findings

The synthesis of findings from the eight included studies is summarized below, grouped by the type of NAI.

Cranberry products: Wing et al. [17] and Wing et al. [18]: These studies investigated cranberry juice and cranberry capsules. The study by Wing (2008) [17] found no statistically significant difference in UTI incidence between the cranberry and placebo groups (p=0.71), while Wing (2015) [18] demonstrated a significant reduction in UTI incidence in the cranberry capsule group (p=0.04). The mixed results may be due to variations in dosage and adherence.

Heitmann [15]: A cohort study on cranberry use reported a higher UTI rate in the cranberry group compared to non-users, but this study had significant limitations, including a high risk of recall bias and a lack of standardization in cranberry dosage, which diminished the reliability of its findings.

Mediterranean diet: Assaf-Balut et al. [11]: The RCT on a Mediterranean diet enhanced with extra virgin olive oil and pistachios showed a statistically significant reduction in UTI incidence in the intervention group (p=0.001). The cohort study by the same author also found that higher adherence to the Mediterranean diet was associated with lower UTI rates. The diet interventions included high levels of vegetables, fruits, nuts, and olive oil, which have well-documented anti-inflammatory and antimicrobial properties [11].

OM-89: Baertschi et al. [19]: The pre-post study on OM-89, a bacterial extract vaccine, found a significant reduction in UTI recurrence after vaccination (p=0.002). However, the study’s pre-post design and lack of randomization contribute to its moderate to high risk of bias, limiting the strength of its conclusions.

Ascorbic acid: Ochoa-Brust et al. [20]: This RCT demonstrated that ascorbic acid supplementation significantly reduced UTI incidence compared to a placebo group (p=0.03). Ascorbic acid’s antimicrobial properties have been well documented, and this study’s results support its potential use in UTI prevention during pregnancy. However, the study's moderate risk of bias due to small sample size and potential issues with blinding of participants suggests that further research is needed.

Knowledge-based interventions: Navarro et al. [12]: A quasi-experimental study was conducted to investigate the impact of knowledge-based interventions on UTI prevention in pregnancy. Participants received educational seminars and text messages covering proper genital hygiene, adequate fluid intake, and regular urination, resulting in a 50% reduction in UTI incidence. However, the high risk of bias in this study, including a lack of control for confounders and incomplete follow-up, limits the generalizability of these findings [12].

Summary of Evidence

Cranberry products: Evidence supports the use of cranberry products, particularly cranberry capsules, in reducing UTI incidence in pregnancy. However, results are mixed, and further studies are needed to confirm these findings, particularly with standardized dosages.

Mediterranean diet: Strong evidence supports the Mediterranean diet as effective in reducing UTI rates during pregnancy, with higher adherence leading to better outcomes. This intervention appears promising but requires further validation in large-scale, randomized trials.

OM-89: OM-89 showed potential in preventing UTI recurrence but requires further investigation, particularly through RCTs, to confirm its safety and effectiveness in pregnant women.

Ascorbic acid: Ascorbic acid supplementation is a promising intervention for preventing UTIs during pregnancy, but more recent studies are needed to evaluate its long-term effectiveness and cost-effectiveness.

Knowledge-based interventions: Educating pregnant women about UTI prevention can significantly reduce UTI incidence, especially in resource-limited settings, though further studies are needed to evaluate its effectiveness in different populations.

Overall, seven of the eight included studies favored the use of NAIs for preventing UTIs during pregnancy. However, the evidence quality is generally low, and further high-quality studies are needed to establish clear guidelines for their use in clinical practice.

Discussion

Key Findings

This systematic review was done to evaluate the effectiveness of NAIs in preventing UTIs during pregnancy as a means to tackle AMR. This review highlights and summarises findings from eight included studies. It was noted from some studies that cranberry products, particularly cranberry capsules, showed a significant reduction in the incidence of UTIs [17,18]. There is sufficient evidence around cranberries’ ability to inhibit bacterial adhesion to the urinary tract, hence proving it to be a preventative intervention for UTIs in pregnancy [10]. However, it was noted that results were inconsistent across the studies, likely due to variations in the formulation of cranberry, dosage, and adherence levels. Data from the included studies showed that the Mediterranean diet, especially when included with olive oil and pistachios, consistently showed significant reductions in UTI incidences amongst pregnant women, due to its anti-inflammatory and antimicrobial properties [11]. It also has broader health benefits, including improved cardiovascular health, and hence is a sustainable and promising intervention for preventing UTIs. OM-89, a vaccine made from bacterial extract, showed promising results in preventing UTI recurrence in a study by Baertschi [19]. However, this requires further research, particularly through RCTs, to confirm its safety and effectiveness in pregnant populations and then further cost-effective studies to measure feasibility.

Ascorbic acid supplements were also found to reduce UTI incidences during pregnancy through their ability to acidify urine and inhibit bacterial growth, proving them to be a preventative intervention [20]. However, the evidence surrounding this is inconclusive and insufficient and requires further research to evaluate its long-term safety during pregnancy. Finally, knowledge-based interventions such as educational interventions (e.g., seminars and text message reminders) showed a significant reduction in UTI incidences during pregnancy [12]. This low-cost intervention was particularly promising in areas where access to healthcare was limited. These strategies may be more effective when integrated into routine antenatal care through guidelines and leaflets, offering an easily accessible preventative measure during pregnancy. While seven out of the eight included studies supported the use of NAIs to prevent/reduce UTI incidence, the overall quality of evidence was low due to significant flaws in the methodologies, risks of bias in the study designs, and inconsistent interventions across the studies.

Evidence of NAIs

There are various studies highlighting the effectiveness of NAIs such as cranberry, the Mediterranean diet, ascorbic acid, and other interventions, but studies specific to pregnant women remain limited. Cranberry capsules have shown potential to prevent/reduce UTI incidence [10]. However, due to variations in cranberry formulation and dosage, the study outcomes are variable, suggesting the need for further standardised trials to determine the best cranberry intervention for pregnant women. The Mediterranean diet, especially when incorporated with extra virgin olive oil and pistachios, has been shown to drastically lower UTI incidence during pregnancy [11], but more research is needed to determine the best diet and adherence.

As shown in Baertschi [19], OM-89 may reduce UTI recurrence, but further research is needed to prove its efficacy and safety during pregnancy. The lack of RCTs and a pre-post study design limits the evidence surrounding OM-89. Ascorbic acid supplementation, which is readily available and affordable, may prevent UTIs during pregnancy [20], but more research is needed to determine its long-term effects during pregnancy and safety at various concentrations. Educating pregnant women on UTI prevention through knowledge-based interventions, which is a low-cost and easily implementable strategy, has shown effectiveness in preventing/reducing UTI incidence [12]. However, more research is needed to explore the optimal educational methods and their long-term impacts. Despite evidence suggesting the promising nature of these NAIs, the evidence remains insufficient and inconclusive due to study limitations, such as small sample sizes, lack of blinding, and inconsistent methodological approaches across the various studies. Therefore, further high-quality studies are necessary to confirm the effectiveness of these NAIs to prevent UTIs during pregnancy.

Strengths and Limitations

Strengths: This systematic review is a comprehensive review that included studies that used a variety of NAIs, offering a broad perspective on interventions other than antibiotics to prevent UTIs in pregnancy as a means to reduce AMR in pregnancy. This review used detailed and standard quality assessment tools such as the Downs and Black tool, the Cochrane Risk of Bias tool, and the CASP checklists, ensuring a thorough evaluation of the included studies and assessing their risk of bias. Unlike many other studies/reviews, this review specifically targeted the pregnant population and addressed a critical gap in existing literature.

Limitations: The quality of evidence was low overall due to considerable bias in non-RCTs. The inferences derived from the findings are affected by these biases. The NAIs were heterogeneous, therefore making it difficult to compare research and make generalised inferences (e.g, cranberry form and dosage, variations in concentration of ascorbic acid, diet composition). Most of the studies included in this review did not capture/assess long-term outcomes, such as recurrence rates or adverse effects on foetal development. Additionally, another limitation of the study is that the review made use of three databases, which may have resulted in missing relevant studies. Furthermore, most of the included studies had small sample sizes and had inconsistent methodological approaches, such as a lack of appropriate randomisation and blinding. Few RCTs were considered, but could not be included due to unclear reporting and methodological flaws.

Implications for Practice

According to this review, of all NAIs, cranberry products and the Mediterranean diet showed the most promising results in reducing/preventing UTIs during pregnancy. Given the rising concern surrounding AMR in pregnancy and the limited number of antibiotics safe to use during pregnancy, these measures may reduce antibiotic consumption and therefore AMR risk. These NAIs should be used as part of clinical practice in antenatal care, especially for pregnant women at higher risk of developing UTIs. However, considering high-quality evidence is scarce, clinical adoption should be cautious, and clinicians should monitor efficacy and maternal and foetal safety when using these NAIs. Large-scale RCTs with standardised interventions are required to strengthen evidence surrounding NAIs and their potential to replace antibiotics during pregnancy.

Future Research Directions

There are several important gaps in the literature surrounding the current evidence on NAIs for preventing UTIs during pregnancy. There is a need for large-scale, high-quality RCTs that compare NAIs to placebo and antibiotics, with clear outcome measures and standardized intervention protocols. The long-term effects and safety surrounding these NAIs need further research (e.g., OM-89), for both pregnant women and their foetus. Future research should compare different NAIs and determine the most effective NAI in preventing UTIs during pregnancy. There is a need for further studies to assess the mechanism of action behind the effectiveness of NAIs, to strengthen evidence, and to better understand these interventions in preventing UTIs. Finally, cost-effective studies are required to evaluate the cost-effectiveness of NAIs compared to antibiotics, as this will be crucial for policymakers when changing guidelines or practice, especially in resource-limited healthcare settings. In conclusion, this review summarises the potential of NAIs as suitable alternatives to antibiotics in preventing UTIs in pregnancy, but there is a need for further rigorous studies to strengthen evidence and establish their efficacy, safety, and feasibility to incorporate in clinical practice.

## Conclusions

This review highlights the benefits of NAIs, including cranberry products, the Mediterranean diet, OM-89, ascorbic acid, and knowledge-based strategies, in UTI during pregnancy. Cranberry capsules and Mediterranean diet interventions demonstrated the most consistent reductions in UTI incidence, likely due to their anti-inflammatory and antimicrobial properties. OM-89 and ascorbic acid also showed promise, while educational and knowledge-based interventions were effective, particularly in resource-limited settings. The clinical and policy implications of these findings are significant. NAIs may offer safe and effective alternatives to antibiotics, help reduce antimicrobial resistance, and complement existing UTI prevention strategies, especially for high-risk pregnant women or in areas with high antibiotic use. Although the RCTs reviewed generally showed lower risk of bias, most studies included in this review had significant methodological limitations, resulting in low overall quality of evidence and warranting caution when interpreting the conclusions. Nevertheless, caution is warranted in their implementation due to the limited and variable evidence. Large-scale, well-designed RCTs are urgently needed to confirm their safety, efficacy, and cost-effectiveness and to establish standardised protocols for clinical practice. Future research should also explore the optimal combination of NAIs, assess their long-term outcomes, and investigate their impact on clinical decision-making, particularly in reducing antibiotic prescribing and enhancing maternal and fetal health. Clinicians may consider integrating NAIs as complementary strategies within existing UTI prevention protocols while closely monitoring patient outcomes.
